# Evolutionary Applications research highlights for issue 9: the ever-evolving field of agriculture

**DOI:** 10.1111/eva.12224

**Published:** 2014-12-09

**Authors:** Britt Koskella

The earliest application of evolutionary theory, although unknowingly at the time, was artificial selection of crops and animals for food production. Ever increasing technical advances in breeding, genetic engineering and comparative genomics have since led to a rapid acceleration in the rate of such selection, although many of the basic principles underlying the process have remained the same over time. For example, whereas we used to inter-breed among genotypes and even species to generate standing genetic variation upon which to select, we can now introduce specific genes of interest directly into the preferred genetic background.

Much of crop domestication historically has involved increased yield and size (for example of fruit or seed), and this has resulted in parallel and often convergent selection upon traits and even genes of interest. Recent work by Dorian Fuller and colleagues used archaeological plant remains from around the world to examine the parallel acquisition of so-called “domestication syndrome traits” across both plant species and regions (Fuller et al. 2014). The authors found differences in the rate of evolution among domestication traits, but also saw remarkably similar rates across regions over periods spanning several centuries to millennia. This work was part of a special feature in PNAS on “the modern view of domestication,” in which 25 researchers from across five fields came together to both discuss progress being made in research on domestication and to identify key challenges for the future (Larson et al. 2014). The feature highlights the role of past domestication in shaping the variation in agricultural species we observe today and suggests future studies should address the role that the contemporary environmental and ecological context may have played in influencing selection on traits in the past.

Improved understanding of the evolutionary process as well as major technological advances means the pace of artificial selection has intensified and our ability to respond to changes in both the abiotic and biotic environment has improved greatly. Our ability to translate understanding of plant genetics and genomics into meaningful applications in crop science is discussed in a new piece by Pamela Ronald (Ronald 2014). The work emphasizes not only the great potential that marker assisted selection, genetic engineering, and genome editing hold in translational research, but also the great need to ensure such technologies benefit farmers in less well-developed countries. Of course the success of newly introduced agricultural varieties will depend on both the local environment and the subsequent evolution of other interacting species. As such, two new papers have focused on the importance of taking into account the evolutionary response of disease agents when guiding disease management practice in agriculture (Burdon et al. 2014; Zhan et al. 2014). For example, Jeremy Burdon and coauthors review the success of strategies such as stacking resistance genes, introducing partial or adult-only resistance, or using mixtures of host types to hinder pathogen evolution (Burdon et al. 2014). Similarly, Jiasui Zhan and collaborators discuss the importance of mimicking the spatial and temporal dynamics of natural host-pathogen coevolution when designing disease management strategies, and emphasize that resistance strategies with immediate short-term benefits are often the least durable in the long term (Zhan et al. 2014).

Given the rapid potential for adaptation, many predictions regarding pest or pathogen evolution can be directly tested in the laboratory in order to inform better disease management. Recent work by Julia Hillung and colleagues examined the adaptation of a plant RNA virus to various ecotypes of *Arabidopsis thaliana* in order to determine the specificity and consequences of evolution on one host to infectivity on another. They use experimental evolution to show rapid increases in infectivity and virulence on the host background in which the virus has been adapted, but also demonstrate that some host types select for viral populations that are more generally infective to other types (Hillung et al. 2014). These results are particularly intriguing in that they suggest manipulation of host types in an agricultural setting could predictably alter the outcome of pathogen evolution. Such rapid evolution is not restricted to the laboratory; evidence from the Western corn rootworm on maize crops indicates that the pest is evolving resistance to the toxins produced by genetically engineered plants that were introduced into production only in 2003 (Gassmann et al. 2014).

Importantly, the utility of evolutionary theory for agricultural practice is not limited to pest and pathogen interactions. The increasingly clear role of the microbiomes across the rhizosphere and phyllosphere suggest great potential for application of both community ecological and evolutionary thinking. Suzanne Donn and coauthors examined the changing soil microbiome of intensive wheat crops across years and found that, relative to soil in the absence of plants, rhizosphere communities changed substantially over time in the presence of plant roots and these temporal dynamics could be explained well based on the stage of plant development (Donn et al. 2014). Such knowledge about tightly coevolved plant-microbe interactions could help inform better management of soils and guide efforts to develop plant probiotics. Another attractive application of evolution to agriculture that has received recent attention is the incorporation of inclusive fitness theory. Toby Kiers and Ford Denison discuss ways in which artificial selection can be focused on improving cooperation among crop plants and the microbial symbionts with which they interact (Kiers and Denison 2014). For example, the authors suggest that the use of those crop types capable of imposing strong sanctions against “cheating” rhizobial bacteria strains (i.e. those that do not fix nitrogen as effectively) could increase the dominance of more mutualistic strains in the soil.

Overall, although artificial selection has been central to agricultural practice since its dawn, we are still constantly improving our ability to speed up the selective process, incorporate adaptation across heterogeneous environments, and allow for a more responsive management program in the face of coevolving enemies and mutualists. As such, there remains great promise in our ability to increase crop yield and decrease the use of pesticides and fertilizers through the application of evolutionary thinking.
